# PIE-1 Translation in the Germline Lineage Contributes to PIE-1 Asymmetry in the Early *Caenorhabditis elegans* Embryo

**DOI:** 10.1534/g3.118.200744

**Published:** 2018-10-02

**Authors:** Timothy J. Gauvin, Bingjie Han, Michael J. Sun, Erik E. Griffin

**Affiliations:** Department of Biological Sciences, Dartmouth College, Hanover NH, 03755

**Keywords:** *C. elegans*, germline, PIE-1, asymmetric cell division, exon junction complex

## Abstract

In the *C. elegans* embryo, the germline lineage is established through successive asymmetric cell divisions that each generate a somatic and a germline daughter cell. PIE-1 is an essential maternal factor that is enriched in embryonic germline cells and is required for germline specification. We estimated the absolute concentration of PIE-1::GFP in germline cells and find that PIE-1::GFP concentration increases by roughly 4.5 fold, from 92 nM to 424 nM, between the 1 and 4-cell stages. Previous studies have shown that the preferential inheritance of PIE-1 by germline daughter cells and the degradation of PIE-1 in somatic cells are important for PIE-1 enrichment in germline cells. In this study, we provide evidence that the preferential translation of maternal PIE-1::GFP transcripts in the germline also contributes to PIE-1::GFP enrichment. Through an RNAi screen, we identified Y14 and MAG-1 (*Drosophila tsunagi* and *mago nashi*) as regulators of embryonic PIE-1::GFP levels. We show that Y14 and MAG-1 do not regulate PIE-1 degradation, segregation or synthesis in the early embryo, but do regulate the concentration of maternally-deposited PIE-1::GFP. Taken together, or findings point to an important role for translational control in the regulation of PIE-1 levels in the germline lineage.

The establishment of the germline lineage is essential for the reproductive success of a developing organism. In the developing embryos of many animals, germ cells are transcriptionally quiescent, which protects them from adopting somatic identities (Seydoux and Braun 2006, Strome and Updike 2015). In the *Drosophila* embryo, the non-coding RNA *polar granule component* (pgc) concentrates in pole cells and represses transcription by inhibiting phosphorylation of Ser2 on the carboxy-terminal domain of RNA polymerase II (Martinho, Kunwar *et al.* 2004). In the 1-cell *C. elegans* embryo, transcription is repressed by OMA-1 and OMA-2, which sequester the TFIID component TAF-4 in the cytoplasm (Guven-Ozkan, Nishi *et al.* 2008). Between the 4-cell and ∼100-cell stages, transcription is repressed in the germline lineage by PIE-1 (Seydoux, Mello *et al.* 1996), which inhibits phosphorylation of the carboxy-terminal domain of RNA polymerase II (Seydoux and Dunn 1997, Batchelder, Dunn *et al.* 1999, Zhang, Barboric *et al.* 2003, Ghosh and Seydoux 2008). In embryos lacking PIE-1 function, the germline blastomere at the 4-cell stage, P2, inappropriately activates transcription (Seydoux, Mello *et al.* 1996) and adopts an identity similar to its somatic sister, EMS, resulting in embryonic lethality (Mello, Draper *et al.* 1992).

Beginning with the division of the 1-cell embryo, the germline lineage is established in a series of four successive asymmetric cell divisions (Rose and Gonczy 2014). Each successive division gives rise to a germline cell (P1, P2, P3 and P4) and a somatic sister cell (Figure 1A). P4 undergoes a single symmetric division to give rise to the primordial germ cells Z2 and Z3, which proliferate to form the germline during larval development (Wang and Seydoux 2013). PIE-1 is maternally deposited in the embryo and is highly concentrated in the P lineage (Mello, Schubert *et al.* 1996, Tenenhaus, Schubert *et al.* 1998). PIE-1 is degraded in Z2 and Z3 around the ∼100-cell stage, which coincides with activation of transcription in these cells (Seydoux and Fire 1994). In addition to its inhibition of transcription in the nucleus, PIE-1 is also present in the cytoplasm where it acts to regulate translation of at least two transcripts, *mom-2 and nos-2* (Oldenbroek, Robertson *et al.* 2013, Tenenhaus, Subramaniam *et al.* 2001).

**Figure 1:**
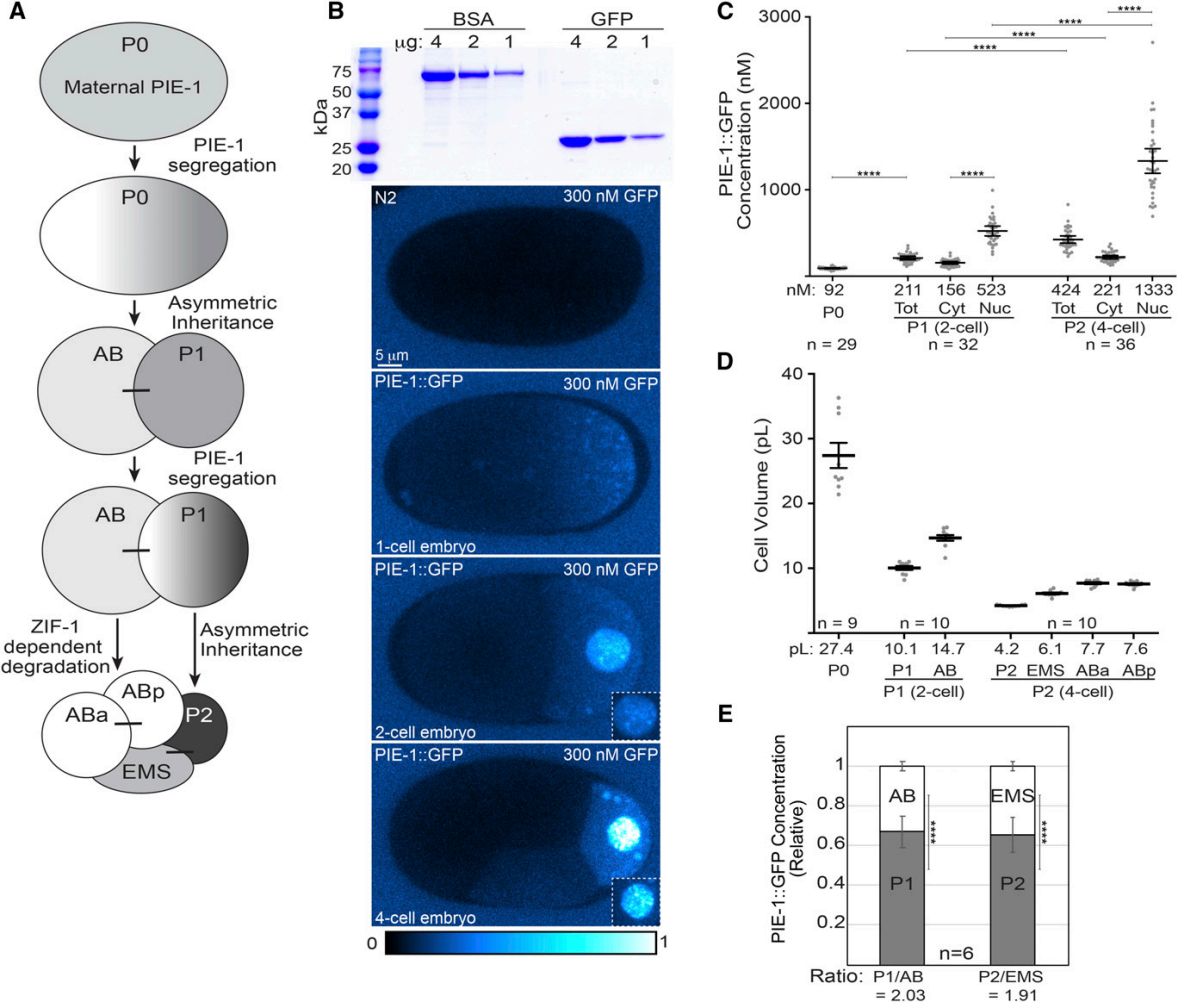
Quantification of the increase in PIE-1::GFP concentration in the P lineage. A. Schematic of PIE-1 (gray) localization from the 1-cell to the 4-cell stage. Maternally deposited PIE-1 segregates asymmetrically to the germline blastomeres P1 and P2 during the first two rounds of cell division. PIE-1 is also degraded in somatic cells. Sister cells are connected by a line. B. Top panel: Coomassie stained SDS-PAGE gel of recombinant GFP and BSA, which was used as a loading standard. Bottom panels: Images of N2 and PIE-1::GFP embryos bathed in 300 nM GFP. Images were pseudocolored using the CyanHot lookup table in ImageJ (scale at the bottom). In order to highlight the dimmer PIE-1::GFP signals, the nuclear signal in the main image of the 4-cell embryo is saturated. The image normalization was adjusted equivalently in the 2 and 4-cell embryo insets such that the nuclear signal is not saturated. PIE-1::GFP concentration in the 1-cell embryo was determined using a 150 nM GFP bath, but is shown in a bath of 300 nM GFP to allow comparison with the later stage embryos. C. Estimates of PIE-1::GFP concentration in P0, P1 and P2. For P1 and P2, concentration estimates are shown for the entire cell (Tot), the cytoplasm (Cyt) and for the nucleus (Nuc). Mean concentrations and the number of embryos analyzed are indicated below the graph. Error bars represent 95% confidence intervals. Statistical significance was determined using unpaired *t*-tests with Welch’s correction for comparisons between embryos (P0 *vs.* P1; P1 *vs.* P2) and using paired *t*-tests for comparisons between cytoplasmic and nuclear concentrations in either P1 or P2. In this and subsequent figures: * = *P* < 0.05, ** = *P* < 0.01, *** = *P* < 0.001, **** = *P* < 0.0001, n.s. = not significant. D. Estimates of the volume of each cell from the 1 to 4-cell stage, determined using embryos expressing GFP::PH^PLCδ1^ (Audhya, Hyndman *et al.* 2005), which marks the plasma membrane. E. The relative concentration of PIE-1::GFP in germline and somatic daughter cells (P1 and AB; P2 and EMS) just after the division of P0 and P1. Statistical significance was determined using an unpaired *t*-test with Welch’s correction. Error bars represent 95% confidence intervals.

Maternally deposited PIE-1 is initially symmetrically distributed in the newly fertilized embryo and becomes progressively enriched in the P lineage as a consequence of two post-transcriptional mechanisms (illustrated in Figure 1A) (Reese, Dunn *et al.* 2000). First, prior to each P cell division, the distribution of PIE-1 becomes polarized such that PIE-1 is preferentially inherited by the P daughter cell (Mello, Schubert *et al.* 1996, Tenenhaus, Schubert *et al.* 1998). In the zygote, the polarization of PIE-1 is controlled by the RNA-binding proteins MEX-5 and MEX-6 (MEX-5/6 hereafter), which segregate to the cytoplasm opposite to PIE-1 (Schubert, Lin *et al.* 2000, Cuenca, Schetter *et al.* 2003). MEX-5/6 control PIE-1 segregation through post-translational mechanisms that inhibit PIE-1 retention in the anterior (Wu, Zhang *et al.* 2015, Han, Antkowiak *et al.* 2018, Wu, Han *et al.* 2018). The second mechanism that contributes to the enrichment of PIE-1 in the P lineage involves the degradation of PIE-1 in somatic cells. During each asymmetric division, the preferential inheritance of PIE-1 by the P daughter cells is not complete, resulting in low levels of PIE-1 inheritance by the somatic daughter cells. In somatic cells, the E3 ubiquitin ligase subunit ZIF-1 binds the first PIE-1 zinc-finger domain and targets PIE-1 for Cullin-dependent degradation (Reese, Dunn *et al.* 2000, DeRenzo, Reese *et al.* 2003). In somatic cells, MEX-5/6 are required for ZIF-1 translation and therefore for PIE-1 degradation (DeRenzo, Reese *et al.* 2003, Oldenbroek, Robertson *et al.* 2012). ZIF-1 translation is repressed in the P lineage, thereby ensuring PIE-1 is only degraded in somatic cells (Oldenbroek, Robertson *et al.* 2012). Like many maternally-deposited mRNAs (Seydoux and Fire 1994), *pie-1* mRNA is present in all blastomeres up to the 4-cell stage and is subsequently degraded in somatic cells and maintained in germline cells (Mello, Schubert *et al.* 1996, Tenenhaus, Schubert *et al.* 1998).

In this study, we present a quantitative analysis of PIE-1::GFP dynamics in the early embryo. We find that PIE-1::GFP translation contributes significantly to its enrichment in the P lineage. In addition, we show that depletion of Y14 and MAG-1 (homologs of *Drosophila* Tsunagi and Mago Nashi, respectively) reduces the concentration of PIE-1::GFP that is maternally deposited in the zygote, resulting in a decrease in the concentration of PIE-1::GFP in the germline blastomere at the 4-cell stage. Taken together, these findings suggest that distinct translational regulation mechanisms during oogenesis and early embryogenesis contribute to the enrichment of PIE-1 in the embryonic germline lineage.

## Materials And Methods

### C. elegans strains

*C. elegans* strains were maintained at 20° on NGM plates seeded with OP50 (Brenner 1974). RNAi feeding was performed at 25°. The following strains were used in this study: N2 (Bristol strain); WM330: *pie-1(ne4301[pie-1*::*GFP])* III (Kim, Ishidate *et al.* 2014); EGD410: pie-1*(ne4301[pie-1*::*gfp])*; *zif-1(egx5*) III; EGD134: *mex-1(egx6[mex-1*::*gfp])* II; JH3209: *mex-6(ax2065[mex-6*::*gfp])* II (Paix, Wang *et al.* 2014); DUP75: *pgl-1(sam33[pgl-1*::*GFP*::*3xFLAG])* IV (Andralojc, Campbell *et al.* 2017); JH3503: *meg-3(ax3054[meg-3*::*megfp])* X (Smith, Calidas *et al.* 2016); and OD58: ltIs38*([pie-1p*::*GFP*::*PH^PLCδ1^]*, *unc-119 (+))* (Audhya, Hyndman *et al.* 2005). The PIE-1::GFP strain WM330 is referred to as wild-type unless otherwise specified.

### Gene editing

Gene editing was performed using the *dpy-10* co-CRISPR approach (Arribere, Bell *et al.* 2014) as described (Paix, Wang *et al.* 2014). sgRNAs were expressed from pRB1017 (Arribere, Bell *et al.* 2014).

#### zif-1(egx5):

The *zif-1(egx5)* allele was isolated in the course of attempting to isolate a deletion of the entire *zif-1* gene. sgRNAs targeting the 5′ (TCTGTGTAAATGAGATACCA; pTG80) and 3′ (TCTGTGTAAATGAGATACCA; pTG88) ends of *zif-1* were used along with an ssODN (TJG203, sequence available upon request; IDT) homology repair template. This ssODN encodes an *Nhe*I restriction site flanked by ∼60bp homology arms targeting the region 5′ and 3′ of the *zif-1* ATG and STOP codon. WM330 hermaphrodites were injected with a mixture consisting of 50 ng/μL pDD162 (Dickinson, Ward *et al.* 2013), 40 ng/μL of both pTG80 and pTG88, 1.2 μM TJG203, 15 ng/μL pJA58 (Arribere, Bell *et al.* 2014), and 300 nM *dpy-10(cn64)* ssODN homology repair template (Arribere, Bell *et al.* 2014).

#### mex-1::gfp:

To tag MEX-1 with GFP endogenously at the C terminus, the sgRNA sequence GTAGGTAGGGGGTGGACGG (pYWP73) was used. A PCR product containing the GFP coding sequence amplified using oligos YW77 (cgtggtacgagaagattttcgggaaaatgacaatgattcaagaggaatcatcgatgggcggtgaagacgacgatgctcacgaagatcattattcgagaagtaaaggagaagaacttttcactggagttg) and YW78 (gtgagaatttggcagatttttaggtaggtaggtagggggtggacggaggaatccagattatttgtatagttcgtccatgccatgtgtaatccc) was used as the repair template. N2 worms were injected with a mixture consisting of 50 ng/μL pDD162, 40 ng/μL pYWP73, 40 ng/μL repair template, 15 ng/μL pJA58 (Arribere, Bell *et al.* 2014) and 300 nM *dpy-10(cn64)* ssODN.

### Cross to test for zygotic transcription of PIE-1::GFP

PIE-1::GFP males were soaked in 1 mM MitoTracker Red (Thermo Fisher Scientific, M7512) diluted in M9 buffer for 2 hr in the dark at 20° in a 24 well dish. Worms were transferred to a fresh NGM plate seeded with OP50 and incubated overnight in the dark. Males were mated to N2 hermaphrodites on an NGM plate spotted with 20 μL of OP50 for 6 hr, at which point hermaphrodites were dissected and mounted onto 3% agarose pads and sealed with VALAP (1:1:1 vaseline, paraffin, and lanolin). Zygotes with MitoTracker Red signal were imaged for >6 hr using DIC and 40% 488 nm laser power, 120 msec exposures, 5 Z slices with 1 μm step size and 10 min intervals. PIE-1::GFP levels were not quantified in this experiment because there was no detectable PIE-1::GFP signal upon visual inspection.

### RNAi screen

Glycerol stocks were streaked from the Ahringer RNAi library (Source BioScience) (Kamath, Fraser *et al.* 2003). 35 RNAi clones that were not recovered from the library were cloned from cDNA or genomic DNA into L4440 (Timmons and Fire 1998). Positive clones were transformed into HT115 (Timmons and Fire 1998) and plated on LB plates + carbenicillin (100 μg/mL) and tetracycline (5 μg/mL). RNAi bacteria were grown in 3 mL LB + carbenicillin (100 μg/mL) for 8 hr, seeded onto NGM plates containing 1 mM IPTG and 25 μg/mL carbenicillin and incubated overnight (Kamath, Fraser *et al.* 2003). For the initial RNAi screen, L4 hermaphrodites were incubated on RNAi plates for 24 hr at 25°. Hermaphrodites were then dissected in M9 buffer and mounted onto 3% agarose pads. Images of 4-cell embryos were collected after all four nuclei were visible and before EMS elongated along ABa. *spn-4(RNAi)* was used as a positive control for each experiment and L4440 (empty vector) RNAi was used as a negative control. At least 5 images were collected and analyzed. The 59 RNAi depletions that significantly affected PIE-1::GFP levels (as determined by Student’s *t*-test) were retested with a minimum of 11 embryos analyzed.

We found that *Y14(RNAi)* and *mag-1(RNAi)* gave more consistent PIE-1::GFP phenotypes when L3 worms were incubated on RNAi plates for 30 hr. Therefore, all analysis subsequent to the initial screen was performed using 30 hr RNAi incubations. RNAi depletions using the MEX-1::GFP strain were incubated for 40 hr at 20° before imaging because this strain had a reduced brood size at 25°.

### Viability Assay

RNAi was performed as described above. Adult worms were placed onto NGM plates and allowed to lay eggs for 2 hr before removal. The next day, the total number of eggs and L1 stage worms were counted.

### Purification of recombinant GFP protein

GFP was cloned into pGEX-KG (Guan and Dixon 1991), which was modified to include a 6xHis-TEV cleavage site (gift from Henry Higgs, Geisel School of Medicine at Dartmouth). The resulting vector, pTG91 (pGEX-KG-TEV-GFP), was transformed into BL21+pLysS (New England Biolabs) and grown to OD_600_ ∼0.8 in 100 mL Terrific Broth at 37° and expressed overnight at 16° following induction with 1 mM IPTG. Bacterial pellets were resuspended in 20 mL extraction buffer (10 mM Tris-HCl pH 8, 500 mM NaCl, 5 mM EDTA, 1 mM DTT, complete protease inhibitors (Roche)), sonicated twice for 15 one-second pulses and centrifuged for 15 min at 17,000 g at 4°. NP40 was added to the supernatant to 0.1% final concentration. The supernatant was flowed over a 2 mL glutathione resin column that had been equilibrated in GST buffer (10 mM Tris-HCl pH 8, 250 mM NaCl, 1 mM EDTA, 1 mM DTT). The column was washed twice with 3 mL GST buffer. AcTEV protease (Thermo Fisher) was added to the column (10 μL) and incubated overnight with rotation at 4°. The column was drained and washed twice. The TEV elution was loaded onto 1 mL Q Fastflow resin (GE Lifesciences) equilibrated in 10 mM Tris-HCl pH 8, 80 mM NaCl, 1 mM EDTA, 1 mM DTT and washed once with the same buffer. GFP was eluted using 10 mM Tris-HCl pH 8, 250 mM NaCl, 1 mM EDTA, 1 mM DTT using 0.5 mL fractions. The second elution was dialyzed overnight at 4° in 10 mM Tris-HCl pH 8, 140 mM NaCl, 1 mM EDTA, 1 mM DTT using SpectraPor7 MWCO 8000 dialysis tubing. The concentration of GFP was determined by Bradford Assay (BioRad) and confirmed on SDS-PAGE gels using a BSA standard.

### Microscopy

All imaging was performed on a Marianas spinning disk confocal microscope (Intelligent Imaging Innovations, Inc. Denver, CO) built around a Zeiss Axio Observer.Z1 microscope. This microscope is equipped with a Plan-Apochromat 40x/1.3 NA oil immersion DIC objective (Zeiss), a Plan-Apochromat 63x/1.4 NA oil immersion DIC objective (Zeiss), an Evolve EMCCD camera (Photometrics), a 50 mW 488 nm solid state laser, a CSU-X1 spinning disk (Yokogawa) and a Phasor photomanipulation unit (Intelligent Imaging Innovations, Inc.). The microscope was controlled using Slidebook software ver 6.0.14 (Intelligent Imaging Innovations, Inc.). For the initial screen, the mean intensity of the PIE-1::GFP fluorescence in the P2 blastomere was determined using ImageJ. Camera background signal was determined for each image using a region outside of the embryo and was subtracted from the P2 value. Measurements were compiled and tested for statistical significance using a two-tailed Student *t*-test in Matlab (version R2016a, MathWorks). Data were graphed using Graph Pad (ver 6.07). Figures were generated using Adobe Illustrator CS6 (ver 16.0.3).

**Figure 2:**
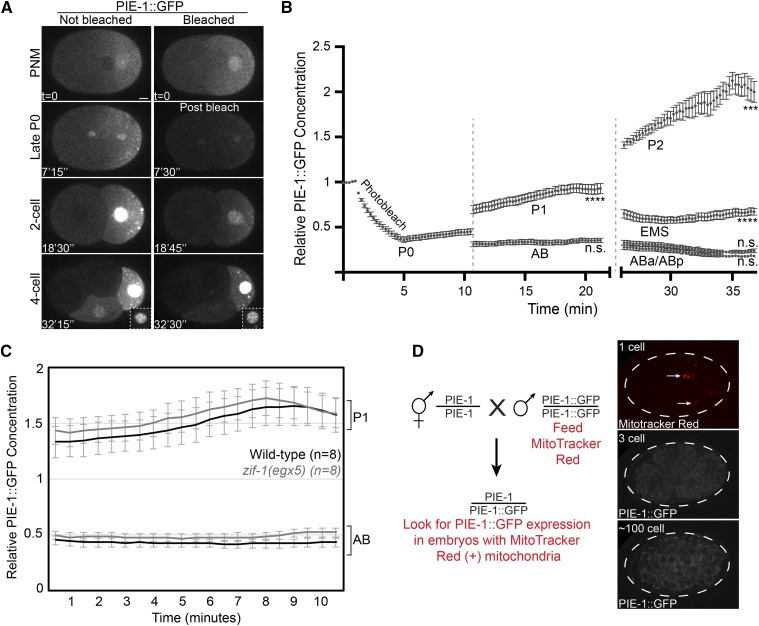
PIE-1::GFP is synthesized in P1 and P2. A. Images of PIE-1::GFP embryos from the 1-cell to the 4-cell stage. PIE-1::GFP fluorescence was bleached in the embryo on the right. Scale bar = 5 μm. PNM, pronuclear meeting in the 1-cell embryo. Time is indicated relative to PNM. B. Quantification of the average PIE-1::GFP fluorescence in embryos that were photobleached at the 1-cell stage. Note the increase in fluorescence in P1 and P2. Values were normalized to the pre-bleach values (n = 6 embryos). Statistical significance (Student’s *t*-test) comparing the final timepoint for each cell to the initial postbleach timepoint is indicated. Error bars represent SEM. Vertical dotted lines indicate cell divisions. C. Change in PIE-1::GFP concentration in P1 and AB in both wild-type and *zif-1(egx5)* embryos. Embryos were imaged following the division of P0. The concentration was normalized to the mean PIE-1::GFP intensity in the entire control embryo. Error bars indicate SEM. D. Zygotic transcription of PIE-1::GFP was tested with the indicated crossing scheme. Cross progeny were identified by the presence of male sperm-derived mitochondria that were labeled with MitoTracker Red (indicated with white arrows in the top panel). No PIE-1::GFP fluorescence was observed in the cross progeny embryos (n = 4).

For FRAP experiments (Figures 2A and 2B), imaging began at pronuclear meeting (PNM) at the cell midplane with 15 sec intervals and ended when ABa and ABp underwent NEBD. After 5 images were collected, an 8 μm diameter circle positioned in the posterior cytoplasm was photobleached for 0.5 sec with 50% laser power and 25 repetitions.

To image changes in PIE-1::GFP levels over time (Figures 2C, 4C, 4D and S1C), the following settings were used: 30% 488 nm laser power, 100 msec exposures and 30 sec intervals. To image PIE-1::GFP beyond the 4-cell stage (Figure 6A), the following settings were used: z-stacks with 1 μm step sizes, 10 min time intervals, 100 msec exposures and 30% 488 nm laser power. To image PIE-1::GFP distribution in adults, hermaphrodites were placed in 5 μL 10 mM levamisole (TCI America) and mounted onto 3% agarose pad. Images were collected with 1000 msec exposures and 55% 488 nm laser power. Two images of the gonad were stitched together using pairwise stitching in FIJI (ImageJ). To determine the posterior:anterior ratio of PIE-1::GFP concentration at NEBD (Figure 4B), the mean intensity of the posterior and anterior halves of the embryo were measured using ImageJ. For quantification of PIE-1::GFP, MEX-1::GFP, and MEX-6::GFP levels, midplane images were collected and analyzed using ImageJ. For all image analysis, camera background was measured outside of the embryo and subtracted from the values inside the embryo.

Blastomere volumes (Figure 1E) were determined using the strain OD58 (Audhya, Hyndman *et al.* 2005), in which GFP localizes to the plasma membrane. 47 Z slices (1 μm step sizes) covering the full embryo volume were collected with 50% 488 nm laser power and 100 msec exposure. Using in Imaris (Bitplane, version 9.1.2, Build 45902), images were segmented using the cell segmentation tool and the cell volume was calculated.

### Estimation of intracellular PIE-1::GFP concentration

Coverslips (Carolina Glass) were washed with 100% xylene for 20-24 hr, acetone for 4 hr, three times with 100% ethanol, once with 75% ethanol and left to dry. Hermaphrodites (N2 or PIE-1::GFP) were dissected in 3 μL of M9 buffer supplemented with 0.1 mg/mL BSA (NEB) and either 300 nM GFP (for 2 and 4-cell embryos) or 150 nM GFP (for 1-cell embryos). Dissected embryos were washed four times with GFP solution. 1 μL containing ∼100 20 μm diameter polystyrene beads in the appropriate GFP bath solution (Bangs Laboratory) was added and the coverslips were sealed to a slide with VALAP.

To correct for uneven illumination, 10 images of GFP solution were collected and averaged. The pixel values of the averaged GFP image were divided into the images of embryos using the image calculator function in ImageJ. The pixel values of the resulting image were then multiplied by the maximum pixel value of the averaged GFP image using the Process > Math > Multiply function in ImageJ. To determine the intracellular GFP concentration, we first corrected the extracellular GFP signal for camera background, which was determined by imaging a solution of M9. We then corrected the intracellular PIE-1::GFP signal for camera background and autofluorescence, which was determined by imaging N2 embryos bathed in GFP solution. The PIE-1::GFP concentration was calculated by multiplying the extracellular GFP concentration by the ratio of the intracellular PIE-1::GFP intensity over the GFP bath intensity.

### Data and Reagent Availability

All strains are available upon request. Strains EGD134 and EGD410 will be deposited at the CGC. Supplemental material available at Figshare: https://doi.org/10.25387/g3.7149386.

## Results

### Quantification of PIE-1::GFP concentration in the early embryo

PIE-1 protein is maternally deposited in the zygote and becomes highly concentrated in the germline lineage (Mello, Schubert *et al.* 1996, Tenenhaus, Schubert *et al.* 1998). We first sought to quantify the changes in PIE-1 concentration that result from the two asymmetric divisions between the 1 and 4-cell stage. We began by estimating the absolute concentration of PIE-1 in each cell between the 1-cell and 4-cell stages. We imaged embryos expressing endogenously-tagged PIE-1::GFP (Kim, Ishidate *et al.* 2014) in a bath of bacterially expressed recombinant GFP, which was used as a standard to calibrate the concentration of PIE-1::GFP in the embryo (Figure 1B). A 150 nM GFP bath was used to estimate the concentration of PIE-1::GFP in the 1-cell embryo and a 300 nM GFP bath was used for 2-cell and 4-cell embryos. Note that the images in Figure 1B show 1, 2 and 4-cell embryos in 300 nM GFP baths to facilitate comparison of PIE-1::GFP levels at the different embryonic stages. Background fluorescence signal was measured within N2 embryos bathed in GFP and was subtracted from the PIE-1::GFP values (Figure 1B). Using this approach, we estimated a mean PIE-1::GFP concentration of 92 nM in the zygote at nuclear envelope breakdown (NEBD) (Figure 1C). We estimated a mean concentration of 211 nM for PIE-1::GFP in the P1 blastomere of 2-cell embryos, with 156 nM in the cytoplasm and 523 nM in the nucleus (Figure 1C). We estimated a mean concentration of 424 nM for PIE-1::GFP in the P2 blastomere of 4-cell embryos, with 221 nM in the cytoplasm and 1333 nM in the nucleus (Figure 1C). These data indicate that the mean concentration of PIE-1::GFP increases ∼4.5 fold in the P lineage between P0 (the zygote) and P2.

One mechanism that contributes to the enrichment of PIE-1::GFP in P2 is the preferential inheritance of PIE-1::GFP by the P daughter during the division of P0 and P1. To determine the extent to which asymmetric inheritance contributes to the ∼4.5 fold increase of PIE-1::GFP concentration, we considered both the difference in the volume of P0 and P2 and the asymmetry in PIE-1::GFP inheritance. We estimated the volume of each cell in the 1, 2 and 4-cell embryo using the Imaris software package to segment and analyze z-stacks of embryos expressing the plasma membrane marker GFP::PH^PLCδ1^ (Audhya, Hyndman *et al.* 2005). We next measured the relative concentration of PIE-1::GFP in the somatic and germline daughter cells immediately following the division of P0 and P1. Following the first division, the ratio of cell volume between P1 and AB is 1:1.46 (10.1:14.7 pL) and the ratio of PIE-1::GFP concentration is 2.03:1 (Figure 1D and 1E). Similarly, following the division of P1, the ratio of cell volume between P2 and EMS is 1:1.45 (4.2:6.1 pL) and the ratio of PIE-1::GFP concentration is 1.91:1 (Figure 1D and 1E). Using these values, we estimate that 58% of PIE-1::GFP segregates to P1 at the first division and 57% segregates to P2 at the second division. Therefore, given an initial PIE-1::GFP concentration of 92 nM, a decrease in volume from P0 to P2 of 6.4 fold, and 58% and 57% efficiency in the segregation of PIE-1::GFP at the first two divisions, respectively, we estimated that the asymmetric inheritance of maternal PIE-1::GFP protein results in a ∼195 nM concentration of PIE-1::GFP in P2, or roughly half of the observed 424 nM concentration (Figure 1C). These findings suggest that in addition to the asymmetric partitioning of maternal PIE-1::GFP, newly synthesized PIE-1::GFP is likely to contribute to PIE-1::GFP levels in P2.

### PIE-1::GFP is synthesized in embryonic germline cells

To test for embryonic synthesis of PIE-1::GFP, we photobleached maternally-contributed PIE-1::GFP in the zygote to ∼40% of its initial fluorescence intensity (Figure 2A). We then measured PIE-1::GFP fluorescence in each cell until the division of ABa and ABp at the 4-cell stage (Figure 2B). We observed that the mean concentration of PIE-1::GFP increased steadily in P0, P1 and P2 (Figure 2B). In the somatic cells, PIE-1::GFP fluorescence either remained constant (AB and EMS) or decreased (ABa and ABp) (Figure 2B). These data are consistent with the possibility that PIE-1::GFP is translated in the germline lineage through the 4-cell stage.

The observation that PIE-1::GFP levels do not increase in somatic blastomeres might be because PIE-1::GFP is not synthesized in somatic cells. Alternatively, the degradation of PIE-1::GFP in somatic cells mediated by the ZIF-1 E3 ubiquitin ligase complex (DeRenzo, Reese *et al.* 2003) could mask PIE-1::GFP synthesis. To distinguish between these possibilities, we measured PIE-1::GFP levels in *zif-1* mutant embryos. We generated a deletion in ZIF-1, *zif-1(egx5)*, which removes 140 bp surrounding the translation start codon (Figure S1) and likely results in a null allele. We found that *zif-1(egx5)* mutant embryos fail to degrade PIE-1::GFP in somatic blastomeres, similar to *zif-1(RNAi)* embryos (Figure S1). Importantly, in both wild-type and *zif-1(egx5)* embryos, there is no increase in PIE-1::GFP levels in AB whereas PIE-1::GFP levels increase in P1 (Figure 2C). Therefore, we conclude that at the 2-cell stage, ZIF-1-dependent degradation does not mask PIE-1::GFP synthesis in AB, consistent with the idea that PIE-1::GFP is translated specifically in P1. Furthermore, the rate of PIE-1::GFP synthesis in P1 is similar in wild-type and *zif-1(egx5)* mutant embryos (Figure 2C), consistent with the previous observations that the ZIF-1-dependent degradation system is not active in the P lineage (DeRenzo, Reese *et al.* 2003).

Embryonic transcription begins in somatic blastomeres at the 4-cell stage and in the germline lineage after the birth of Z2/Z3 (Seydoux, Mello *et al.* 1996). Therefore, we considered it unlikely that PIE-1::GFP transcription in the early embryo is required for embryonic PIE-1::GFP translation. To formally test this possibility, we crossed PIE-1::GFP males to wild-type hermaphrodites (N2 strain in which PIE-1 is not tagged), which do not contribute maternal PIE-1::GFP protein or mRNA to the embryo. PIE-1::GFP males were stained with MitoTracker Red to label sperm mitochondria, which was used to identify cross progeny (schematized in Figure 2D). As expected, we did not detect any PIE-1::GFP signal in cross progeny embryos (n = 4), which were imaged from the 2-cell stage until after the birth of Z2 and Z3 (Figure 2D). We conclude that new synthesis of PIE-1::GFP results from the translation of maternal PIE-1::GFP transcripts that are deposited in the embryo and not from embryonic transcription of PIE-1::GFP.

The above findings indicate that PIE-1::GFP is translated specifically in the P lineage and that this translation contributes significantly to the enrichment of PIE-1::GFP in P2. Specifically, we estimate that roughly half of PIE-1::GFP in P2 is derived from the partitioning of PIE-1::GFP that is translated in the adult germline and deposited maternally into the embryo. We propose that the additional ∼50% of PIE-1::GFP in P2 derives from PIE-1::GFP that is newly synthesized in the embryo. We note that our estimate of the levels of newly synthesized PIE-1::GFP in P2 is likely an underestimate because newly translated PIE-1::GFP needs to fold and mature before it becomes fluorescent. In summary, PIE-1::GFP enrichment in the P lineage results from the combined effects of three mechanisms: the asymmetric segregation of PIE-1::GFP, the degradation of PIE-1::GFP in somatic cells and the translation of PIE-1::GFP in the P lineage.

### RNAi screen for regulators of PIE-1::GFP levels in P2

**Figure 3:**
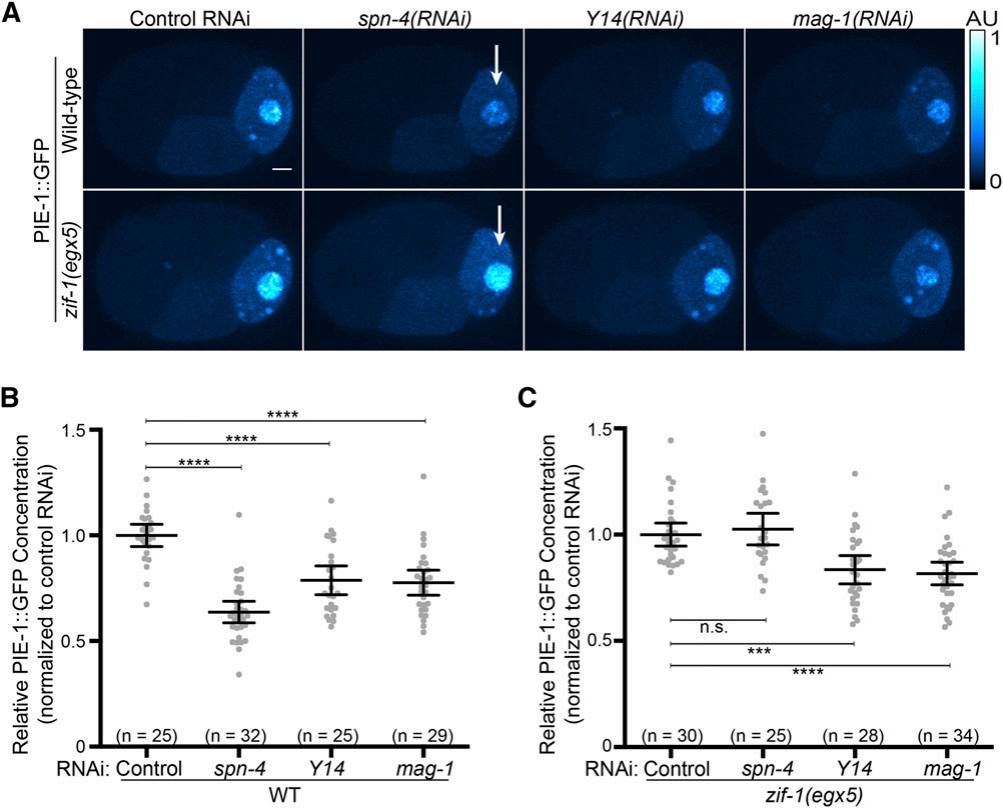
PIE-1::GFP concentration is reduced in P2 in *spn-4(RNAi)*, *Y14(RNAi)*, and *mag-1(RNAi)* embryos. A. Images of PIE-1::GFP in 4-cell embryos of the indicated genotypes. Note that the decrease in PIE-1::GFP levels in spn-4(RNAi) embryos depends on ZIF-1 (indicated by white arrows). Images were pseudocolored using the CyanHot lookup table in ImageJ (scale at the right). Scale bar = 5 µm. B, C. Mean PIE-1::GFP concentration in P2 in wild-type (panel B) and *zif-1(egx5)* (panel C) embryos treated with the indicated RNAi. All values were normalized to the mean of the control RNAi. Error bars indicate 95% confidence intervals. Statistical significance was determined using a Student's t test.

In adult hermaphrodites, PIE-1 translation is repressed in the distal gonad and increases around the bend where oocytes begin to form (Tenenhaus, Schubert *et al.* 1998). The PIE-1 3′ UTR is sufficient to recapitulate this expression pattern (Merritt, Rasoloson *et al.* 2008). These observations along with the finding that PIE-1::GFP is translated in the P lineage highlight the central role of translational regulation in establishing the PIE-1 expression pattern. To begin to characterize the mechanisms that control PIE-1 translation, we performed an RNAi screen of 249 genes encoding proteins with predicted RNA-binding domains commonly found in translational regulators (Tamburino, Ryder *et al.* 2013). We compared the mean concentration of PIE-1::GFP in P2 of RNAi treated embryos relative to control embryos (n ≥ 5 embryos). From this initial screen of all 249 RNAi clones, 59 RNAi clones significantly altered PIE-1::GFP concentration. Upon rescreening with a minimum of 11 embryos quantified (average of 20 embryos), 18 RNAi clones significantly altered PIE-1::GFP concentration (Figure S2). Of these rescreened clones, *spn-4(RNAi)*, *mex-5(RNAi)*, and *Y14(RNAi)* most significantly reduced PIE-1::GFP levels in P2 (Figure 3A, 3B and S2). The identification of MEX-5 (Schubert, Lin *et al.* 2000) and SPN-4 (Ogura, Kishimoto *et al.* 2003) was not surprising. MEX-5 regulates many aspects of the germline/soma dichotomy, including both the segregation and degradation of PIE-1 (Schubert, Lin *et al.* 2000, DeRenzo, Reese *et al.* 2003). SPN-4 is required to repress ZIF-1 translation in the P lineage (Oldenbroek, Robertson *et al.* 2012), suggesting that de-repression of ZIF-1 in the P lineage might account for the reduction in PIE-1::GFP levels in *spn-4(RNAi)* embryos. Consistent with this idea, we find that the reduction in PIE-1::GFP concentration in *spn-4(RNAi)* embryos is dependent on ZIF-1 (Figure 3A and 3C).

We therefore focused our further analysis on Y14, which has not previously been implicated in the regulation of PIE-1 levels. Y14 (also referred to as RNP-4 in *C. elegans*) is a homolog of *Drosophila tsunagi* and mammalian Y14 (Hachet and Ephrussi 2001, Mohr, Dillon *et al.* 2001, Kawano, Kataoka *et al.* 2004). Along with its binding partner Mago Nashi (Newmark and Boswell 1994), Y14 is a component of the exon junction complex (EJC). EJCs are deposited on transcripts during splicing in the nucleus and subsequently regulate diverse aspects of mRNA regulation including nuclear export, stability, transport, nonsense-mediated decay and translation (Le Hir, Sauliere *et al*. 2016). For example, in *Drosophila*, EJC components are essential for the transport of *oskar* mRNA and for the subsequent posterior localization of the germ plasm (Micklem, Dasgupta *et al.* 1997, Newmark, Mohr *et al.* 1997, Hachet and Ephrussi 2004). In *C. elegans*, Y14 interacts with the homolog of Mago Nashi, MAG-1 (Kawano, Kataoka *et al.* 2004, Li, Armstrong *et al.* 2004). Y14 and MAG-1 prevent the nuclear export of unspliced transcripts, are required for embryonic development (Kawano, Kataoka *et al.* 2004, Shiimori, Inoue *et al.* 2013) and contribute to the localization of the P granule marker PGL-1::GFP (Updike and Strome 2009).

**Figure 4:**
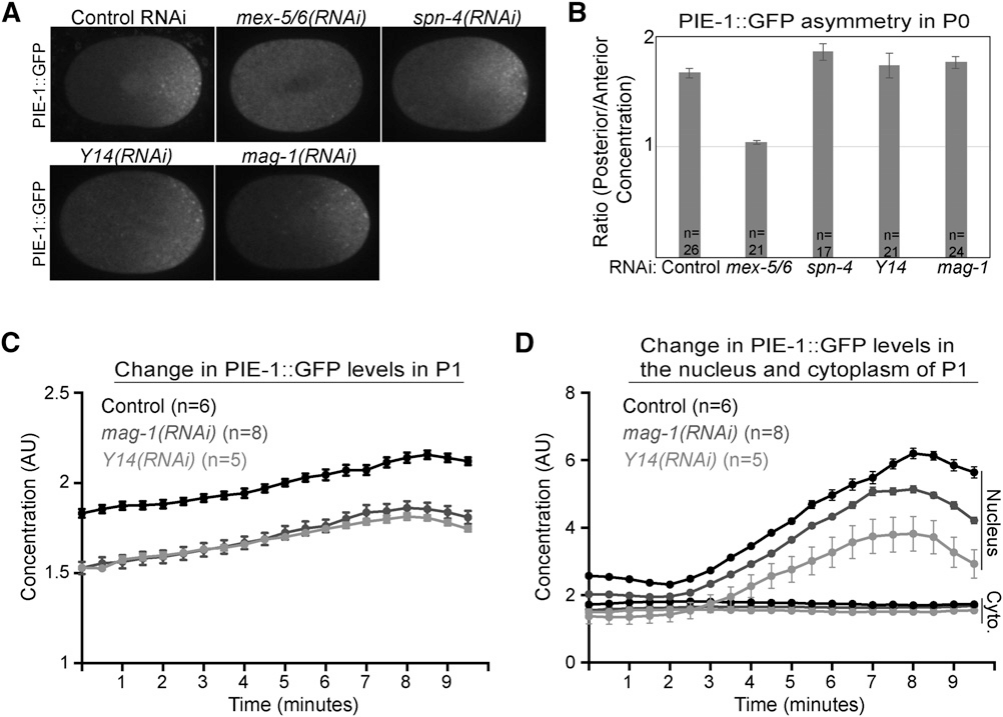
PIE-1::GFP segregation and synthesis in *Y14(RNAi)* and *mag-1(RNAi)* embryos. A. PIE-1::GFP localization in polarized zygotes of the indicated genotype. B. The ratio of PIE-1::GFP concentration in posterior and anterior cytoplasm of zygotes of the indicated genotype at nuclear envelope breakdown. C. Change in the average PIE-1::GFP concentration in P1 in embryos of the indicated genotype. The concentration values were normalized to the entire control embryo, including both AB and P1. D. Change in the average cytoplasmic and nuclear PIE-1::GFP concentration in P1 in embryos of the indicated genotype. The same embryos were analyzed in panels C and D. Error bars indicate SEM in panels B, C and D.

MAG-1 was not among the genes in our initial RNAi screen. We therefore tested whether MAG-1 regulates PIE-1::GFP levels similar to Y14. In *mag-1(RNAi)* embryos, PIE-1::GFP concentration is reduced in P2 to a similar extent as in *Y14(RNAi)* embryos (Figure 3A and 3B). Unlike *spn-4(RNAi)*, both *Y14(RNAi)* and *mag-1(RNAi)* significantly reduced P2 PIE-1::GFP levels in *zif-1(egx5)* embryos (Figure 3C), indicating that the reduction in PIE-1::GFP levels was not due to increased ZIF-1 activity in the P lineage of *Y14(RNAi)* and *mag-1(RNAi)* embryos. In addition, the segregation of PIE-1::GFP to the posterior in *Y14(RNAi)* and *mag-1(RNAi)* 1-cell embryos was similar to wild-type (Figure 4A and 4B). We next asked whether Y14 and MAG-1 are required for PIE-1::GFP synthesis in the embryo. We found that the rate at which PIE-1::GFP levels increased in P1 was similar in *Y14(RNAi)*, *mag-1(RNAi)* and control embryos (Figure 4C and 4D). We noted that the initial concentration of PIE-1::GFP in P1 was lower in *Y14(RNAi)* and *mag-1(RNAi)* embryos (Figure 4C), suggesting that the reduction in PIE-1::GFP levels preceded the 2-cell stage. Taken together, these data indicate that the reduction in PIE-1::GFP concentration in P2 of *Y14(RNAi)* and *mag-1(RNAi)* embryos is not due to derepression of ZIF-1 in germline blastomeres, PIE-1::GFP segregation defects in the zygote or decreased embryonic PIE-1::GFP synthesis.

### Y14 and MAG-1 regulate maternal PIE-1::GFP synthesis

**Figure 5:**
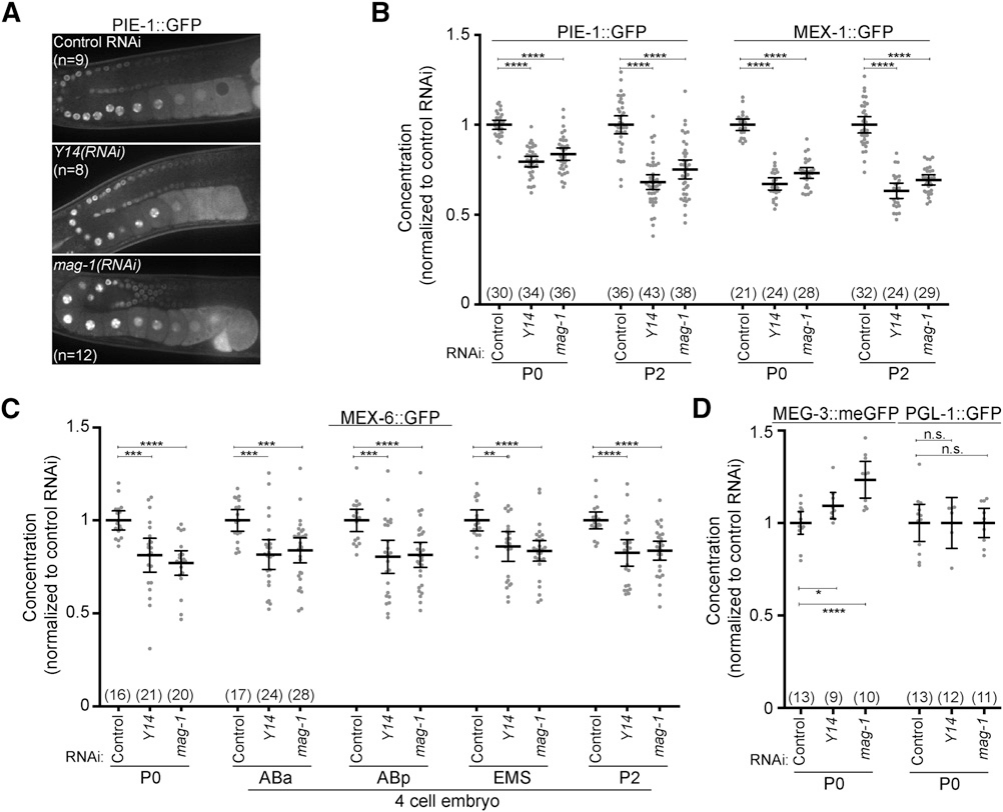
Quantification of the levels of maternally deposited proteins in *Y14(RNAi)* and *mag-1(RNAi)* embryos. A. PIE-1::GFP expression in the germline of adult hermaphrodites treated with the indicated RNAi. B. PIE-1::GFP and MEX-1::GFP fluorescence in P0 and P2 of embryos treated with the indicated RNAi. Values were normalized to the control at both stages. C. MEX-6::GFP fluorescence in 1 and 4-cell embryos treated with the indicated RNAi. Values were normalized to the control for each cell. D. MEG-3::meGFP and PGL-1::GFP fluorescence in 1-cell embryos treated with the indicated RNAi. Values were normalized to the control for each cell. For panels B – D, the number of embryos is indicated in parentheses and error bars indicate 95% confidence intervals.

Y14 and MAG-1 are expressed in oocytes (Kawano, Kataoka *et al.* 2004), suggesting that they could act in the adult germline to regulate the concentration of maternally-deposited PIE-1::GFP. The pattern of PIE-1::GFP expression is similar in the gonads of *Y14(RNAi)*, *mag-1(RNAi)* and wild-type adults (Figure 5A), suggesting Y14 and MAG-1 do not regulate the spatial patterning of PIE-1::GFP expression in the adult germline. To determine whether Y14 and MAG-1 regulate the levels of maternally deposited PIE-1::GFP, we compared the levels of PIE-1::GFP in wild-type, *Y14(RNAi)* and *mag-1(RNAi)* zygotes. We found that PIE-1::GFP levels were significantly lower in both *Y14(RNAi)* and *mag-1(RNAi)* zygotes compared to control zygotes (Figure 5B). We conclude that Y14 and MAG-1 depletion reduces the concentration of maternally-deposited PIE-1::GFP and propose that this reduction results in reduced PIE-1::GFP concentration in the P2 blastomere.

To test the specificity of Y14 and MAG-1 in regulating PIE-1::GFP maternal synthesis, we measured concentrations of endogenously-tagged MEX-1::GFP and MEX-6::GFP at the 1-cell and 4-cell stages. Both proteins have tandem zinc finger domains similar to PIE-1::GFP. MEX-1::GFP concentrates in germline blastomeres similar to PIE-1 (Guedes and Priess 1997) whereas MEX-6 concentrates in somatic blastomeres at the 4-cell stage (Schubert, Lin *et al.* 2000, Cuenca, Schetter *et al.* 2003). In *Y14(RNAi)* and *mag-1(RNAi)* embryos, MEX-1::GFP and MEX-6::GFP concentration were reduced at the 1-cell stage and this reduction persisted to the 4-cell stage (Figure 5B and 5C). In contrast, the concentration of PGL-1::GFP::3xFLAG (Andralojc, Campbell *et al.* 2017) and MEG-3::meGFP (Smith, Calidas *et al.* 2016) did not change and increased slightly, respectively, in *Y14(RNAi)* and *mag-1(RNAi)* zygotes relative to control zygotes (Figure 5D). Interestingly, *Y14(RNAi)* and *mag-1(RNAi)* reduces the expression of transgenic GFP::PGL-1 whose expression is controlled by the PIE-1 promoter and the PIE-1 3′ UTR (Updike and Strome 2009). These results are consistent with the possibility that Y14 and MAG-1 might regulate expression through the PIE-1 promoter and/or the PIE-1 3′ UTR. We conclude that Y14 and MAG-1 regulate the concentration of a subset of maternally-deposited proteins that includes PIE-1::GFP.

### PIE-1::GFP localization After the 4-cell stage in *Y14(RNAi)* and *mag-1(RNAi)* embryos

**Figure 6:**
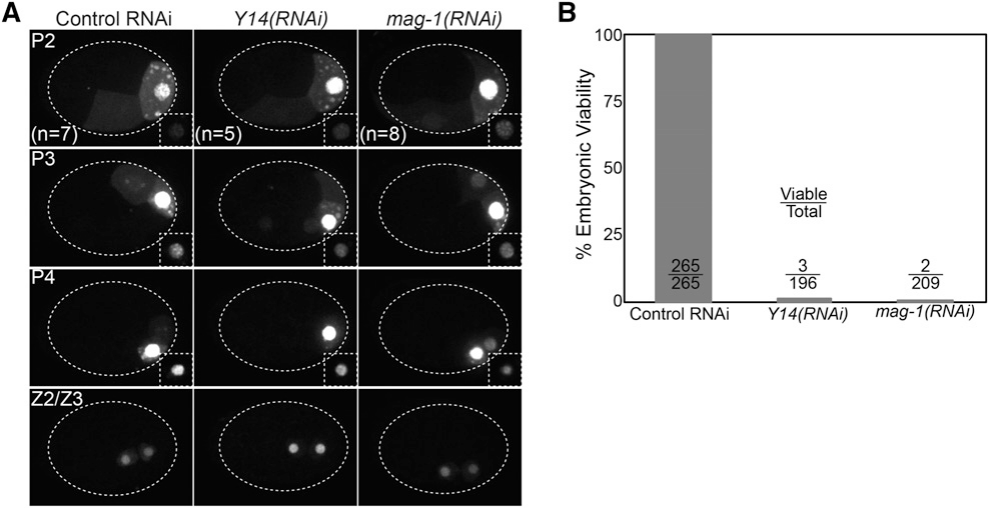
Effects of *Y14(RNAi)* and *mag-1(RNAi)* on PIE-1::GFP localization and embryonic viability. A. PIE-1::GFP localization from the 4-cell to ∼100-cell stage. The nuclear PIE-1::GFP levels are saturated in the main images. Each of the insets in the bottom right corner are normalized equivalently such that the nuclear signals are not saturated. The dotted ellipses outline the embryos. B. Embryonic viability of embryos of the indicated genotype. Error bars indicate SEM.

After the 4-cell stage, the germline lineage undergoes two more asymmetric division and a single symmetric division, giving rise to the primordial germ cells Z2 and Z3, at which point PIE-1 is degraded. The asymmetric segregation and subsequent degradation of PIE-1::GFP following the 4-cell stage appeared normal in both *Y14(RNAi)* and *mag-1(RNAi)* embryos (Figure 6A). Furthermore, the positioning of the P cells and Z2/Z3 appeared normal in *Y14(RNAi)* and *mag-1(RNAi)* embryos. Therefore, the reduction in maternal PIE-1::GFP levels do not appear to cause mislocalization of PIE-1::GFP to cells outside of the P lineage or to alter the division pattern that generates Z2 and Z3. We were not able to assess whether adults derived from *Y14(RNAi)* and *mag-1(RNAi)* embryos displayed germline defects because depletion of either Y14 (Kawano, Kataoka *et al.* 2004) or MAG-1 results in embryonic lethality due to developmental arrest during morphogenesis (Figure 6B).

## Discussion

Translational regulation controls the expression of several maternal transcripts and is essential for patterning the early *C. elegans* embryo. Some transcripts are translated specifically in somatic cells, including *neg-1* (Elewa, Shirayama *et al.* 2015), *zif-1* (Oldenbroek, Robertson *et al.* 2012) and *glp-1* (Ogura, Kishimoto *et al.* 2003). Other transcripts are translated in germline cells, including *mom-2* (Oldenbroek, Robertson *et al.* 2013), *nos-2* (D’Agostino, Merritt *et al.* 2006, Jadhav, Rana *et al.* 2008) and *apx-1* (Tabara, Hill *et al.* 1999). In this study, we provide evidence that PIE-1::GFP is translated specifically in germline cells and not in somatic cells of the early embryo. We propose that PIE-1::GFP translation is a third mechanism that, along with the preferential inheritance by germline cells and degradation in somatic cells, contributes to dramatic enrichment of PIE-1 in the P lineage.

### How might PIE-1 translation be restricted to the P lineage?

Studies of other differentially translated mRNAs in the early embryo have demonstrated the important role of combinatorial regulation by multiple maternal RNA-binding proteins (Oldenbroek, Robertson *et al.* 2013, D’Agostino, Merritt *et al.* 2006, Jadhav, Rana *et al.* 2008, Oldenbroek, Robertson *et al.* 2012). For example, maternal *mom-2* RNA, which encodes the WNT ligand MOM-2, is translated specifically in P2 due to the combined regulation of PIE-1, MEX-5, MEX-6, POS-1, MEX-1, SPN-4 and MEX-3 (Oldenbroek, Robertson *et al.* 2013). MEX-5/6, SPN-4 and MEX-3 repress translation of the *mom-2* mRNA in somatic blastomeres where their levels are high. The levels of these proteins are lower in P2 where the concentration of POS-1 is relatively high, leading to the derepression of *mom*-2 mRNA translation (Oldenbroek, Robertson *et al.* 2013). Interestingly, although SPN-4 and POS-1 are required to pattern the translation of many mRNAs, including *nos-2* (D’Agostino, Merritt *et al.* 2006, Jadhav, Rana *et al.* 2008), *zif-1* (Oldenbroek, Robertson *et al.* 2012), *mom-2* (Oldenbroek, Robertson *et al.* 2013), *glp-1* (Ogura, Kishimoto *et al.* 2003), *apx-1* (Tabara, Hill *et al.* 1999) and *neg-1* (Elewa, Shirayama *et al.* 2015), they do not appear to directly regulate PIE-1::GFP translation before the 4-cell stage. Depletion of POS-1 did not significantly reduce PIE-1::GFP levels in P2 (Figure S2) and depletion of SPN-4 did not significantly increase PIE-1::GFP levels in somatic blastomeres in *zif-1* mutant embryos (Figure 3C).

MEX-5/6 are candidate repressors of PIE-1 translation in somatic cells. Although PIE-1::GFP levels appear qualitatively higher in *mex-5/6(RNAi)* embryos, testing whether MEX-5/6 directly regulate PIE-1 translation is complicated by the fact that MEX-5/6 are broadly required to establish cytoplasmic asymmetries during the early embryonic divisions (Schubert, Lin *et al.* 2000). An additional possibility is that PIE-1 could engage in a positive feedback loop by promoting the translation of *pie-1* mRNA in the P lineage. Consistent with this possibility, PIE-1 promotes the translation of *mom-2* mRNA in P2 (Oldenbroek, Robertson *et al.* 2013) and of *nos-2* mRNA in P4 (Tenenhaus, Subramaniam *et al.* 2001). After the 4-cell stage, the degradation of *pie-1* mRNA in somatic cells (Tenenhaus, Schubert *et al.* 1998) ensures that any PIE-1 translation at later stages will be confined to the germline lineage.

MEX-5, SPN-4 and Y14 were the only RNAi clones that decreased PIE-1::GFP levels by more than 10% in our screen. There are several reasons why our screen might not have identified regulators of PIE-1::GFP translation in the early embryo. Our screen would not have identified genes that act redundantly or were not depleted efficiently using our RNAi procedure. In addition, because we only screened a subset of candidate RNA-binding proteins, it is possible that regulators of embryonic PIE-1::GFP translation were not tested in our screen. In the future, it will be important to identify and characterize the factors that regulate PIE-1 translation in the early embryo and to assess the functional significance of this regulation in specifying the germline lineage.

### Translation regulation by the EJC complex

Through our screen for regulators of PIE-1::GFP levels in P2, we identified the exon junction components Y14 and MAG-1. Components of the EJC are deposited on transcripts in the nucleus during splicing and remain associated as the transcripts are exported to the cytoplasm. The EJC complex provides a docking site for a number of secondary regulatory factors and can regulate diverse processes including mRNA splicing, export, transport, non-sense mediated decay and translational regulation (Le Hir, Sauliere *et al.* 2016). It has been demonstrated that depletion of Y14 results in the leakage of several unspliced transcripts into the cytoplasm in *C. elegans* (Shiimori, Inoue *et al.* 2013). Whether there is leakage of unspliced *pie-1*::*gfp* into the cytoplasm in *Y14(RNAi)* and *mag-1(RNAi)* worms and whether such leakage accounts for the decreased concentration of maternal PIE-1::GFP is not known at this point. Additionally, we note that because depletion of EJC components is likely to alter the expression of many proteins in the adult gonad, the effects on maternally deposited PIE-1::GFP could be indirect.

Mago Nashi and Tsunagi are required for the assembly of germ plasm at the posterior pole of the *Drosophila* oocyte (Newmark and Boswell 1994, Mohr, Dillon *et al.* 2001). Germ plasm assembly is nucleated by *osk* mRNA (Lehmann 2016), which assembles with EJC components into complexes that are transported on microtubules to the posterior (Hachet and Ephrussi 2004, Zimyanin, Belaya *et al.* 2008). *osk* mRNA transport requires the splicing of intron 1 (Hachet and Ephrussi 2004), consistent with the idea that EJC components are deposited on *osk* mRNA during splicing. Mutations in *tsunagi* and *mago nashi* prevent *osk* mRNA localization to the posterior and the subsequent formation of germ cells (Micklem, Dasgupta *et al.* 1997, Newmark, Mohr *et al.* 1997, Hachet and Ephrussi 2001, Mohr, Dillon *et al.* 2001). Our data suggest that EJC components play a more subtle and less direct role in PIE-1 localization in *C. elegans*. This difference may reflect the fact that directed mRNA transport is essential for germ plasm segregation to the posterior in *Drosophila* but is not thought to contribute to the segregation of germ plasm in the *C. elegans* embryo.

In summary, our findings indicate that translational regulation of PIE-1 plays an important role in the dramatic enrichment of PIE-1 in embryonic germ cells. Interestingly, unlike most of the previously described targets of translation regulation in the early embryo, PIE-1 is itself a translation regulator. In the future, it will be interesting to learn whether translational control contributes to the asymmetric distribution of other translational regulators, such as POS-1 and MEX-5. Consistent with this possibility, POS-1 binds to the *mex-6* 3′ UTR and has been proposed to regulate MEX-6 translation (Tenlen, Schisa *et al.* 2006). Such regulation could amplify or refine asymmetries that are established through post-translational mechanisms, thereby reinforcing the specification of discrete embryonic cell fates in the early embryo.
